# Castleman’s disease in the retroperitoneal space mimicking a paraspinal schwannoma: a case report

**DOI:** 10.1186/1477-7819-11-108

**Published:** 2013-05-23

**Authors:** Satoshi Nagano, Masahiro Yokouchi, Takuya Yamamoto, Hideyasu Kaieda, Takao Setoguchi, Tsubasa Hiraki, Yukie Tashiro, Suguru Yonezawa, Setsuro Komiya

**Affiliations:** 1 Department of Orthopedic Surgery, Graduate School of Medical and Dental Sciences, Kagoshima University, 8-35-1 Sakuragaoka, Kagoshima-city, Kagoshima, 890-8520, Japan; 2 Department of Human Pathology, Graduate School of Medical and Dental Sciences, Kagoshima University, 8-35-1 Sakuragaoka, Kagoshima-city, Kagoshima, 890-8520, Japan; 3 The Near-Future Locomotor Organ Medicine Creation Course (Kusunoki Kai), Graduate School of Medical and Dental Sciences, Kagoshima University, 8-35-1 Sakuragaoka, Kagoshima-city, Kagoshima, 890-8520, Japan; 4 Department of Pathology, Imakiire General Hospital, 4-16 Shimotatsuocho, Kagoshima-city, Kagoshima, 892-0852, Japan

**Keywords:** Castleman’s disease, Dumbbell shape, Paraspinal schwannoma, Soft-tissue sarcoma

## Abstract

**Background:**

Castleman’s disease is a rare disease characterized by lymph node hyperplasia. Its occurrence in the retroperitoneal space has rarely been reported, making its preoperative diagnosis difficult. Here, we report a case of retroperitoneal Castleman’s disease, which radiologically resembled paraspinal schwannoma.

**Case presentation:**

A 33-year-old Japanese man with epigastric discomfort underwent abdominal ultrasonic examination revealing a solid mass next to the right kidney. Computed tomography demonstrated a well-circumscribed mass with central calcification in the right psoas muscle. Because the mass presented a dumbbell-like shape extending to the intervertebral foramen, neurogenic tumor was suspected. Both iodine-123 metaiodobenzylguanidine and gallium-67 scintigraphies were negative in the mass, whereas thallium-201 mildly accumulated in the tumor, suggesting blood flow to the tumor. Positron emission tomography revealed accumulation of fluorine-18-2-fluoro-2-deoxy-d-glucose in the tumor at a standard uptake value of 4.7, whereas no other abnormal uptake suggestive of metastatic lesion was noted. On the basis of imaging studies, we mostly suspected paraspinal schwannoma, although malignancy was not completely excluded. Angiography showed feeding vessels from the right lumbar arteries, which were embolized with porous gelatin particles in order to reduce intraoperative bleeding. Surgical resection was performed using a retroperitoneal approach, which revealed the tumor in the swollen psoas muscle. Intraoperative pathological examination of a frozen section revealed no evidence of malignancy; thus, marginal excision of the tumor was performed. The tumor adhered tightly to surrounding muscle tissues, resulting in 940 g of intraoperative blood loss. The pathological examination demonstrated infiltration of lymphocytes surrounding small germinal centers with extensive capillary proliferation. Immunostaining revealed that proliferated lymphocytes were CD3-negative and CD79a-positive.

**Conclusions:**

Although a dumbbell-shaped mass in a paraspinal region is indicative of a schwannoma for orthopedic surgeons, the possibility of Castleman’s disease should be considered if a central low-signal area in fissured and a radial pattern is detected on computed tomography or magnetic resonance imaging. Appropriate preparation for massive bleeding during the treatment of Castleman’s disease, including angiography and embolization, would be helpful for performing surgical procedures safely.

## Background

Castleman’s disease (CD) is a rare disease characterized by lymph node hyperplasia with polyclonal proliferation, first reported by Castleman in 1954 [1]. CD is classified into unicentric CD (UCD) and multicentric CD (MCD) affecting a single lymphatic organ and multiple sites throughout the body, respectively [2,3]. Although MCD is less common, it accompanies systemic chronic inflammatory symptoms induced by interleukin (IL)-6 from affected lymph nodes [3]. MCD carries a worse prognosis, although IL-6 blockade by the recently approved tocilizumab is a promising treatment option [4]. On the other hand, UCD usually involves benign manifestations and can be cured by surgical excision. Although the most common site of UCD is the mediastinum (70%) [5], it may occur anywhere along the lymphatic chain. UCD in the retroperitoneal space is relatively rare, accounting for 6.5–8.2% of all UCD cases [6]. Because of its rarity, the preoperative diagnosis of retroperitoneal UCD is very difficult [7]. Here, we report on a case of retroperitoneal UCD, which resembled radiologically paraspinal schwannoma.

## Case presentation

**Figure 1:**
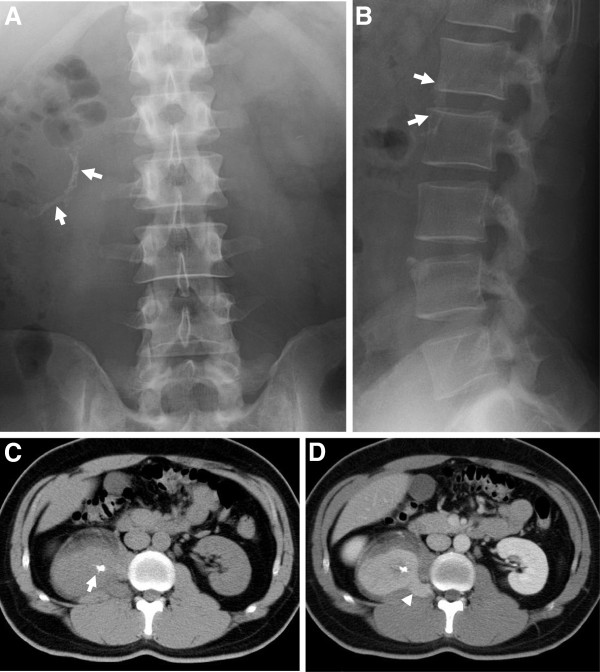
**Plain radiography and computed tomography.** A striated calcification on the right side of the first and second lumbar vertebrates was noted (arrow) in abdominal plain radiography (**A**,**B**). Computed tomography revealed a homogenous mass with central calcification (arrow) in the swollen psoas muscle (**C**). After contrast enhancement, the mass presented a dumbbell-like shape, extending to the intervertebral foramen (**D**).

**Figure 2:**
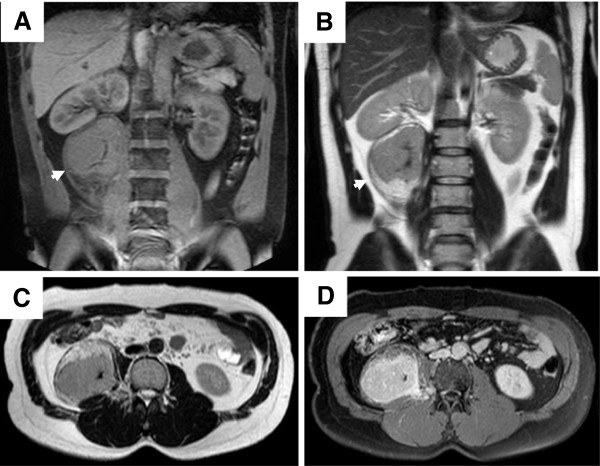
**Magnetic resonance imaging.** The mass presented isointensity on the T1-weighted image (**A**) and hyperintensity on the T2-weighted image (**B**) with a central radial hypointense area. On the caudal and anterior side of the tumor, a lipomatous component was observed on T2-weighted images (**B**,**C**). The contrast-enhanced image showed marked enhancement of the tumor (**D**).

**Figure 3:**
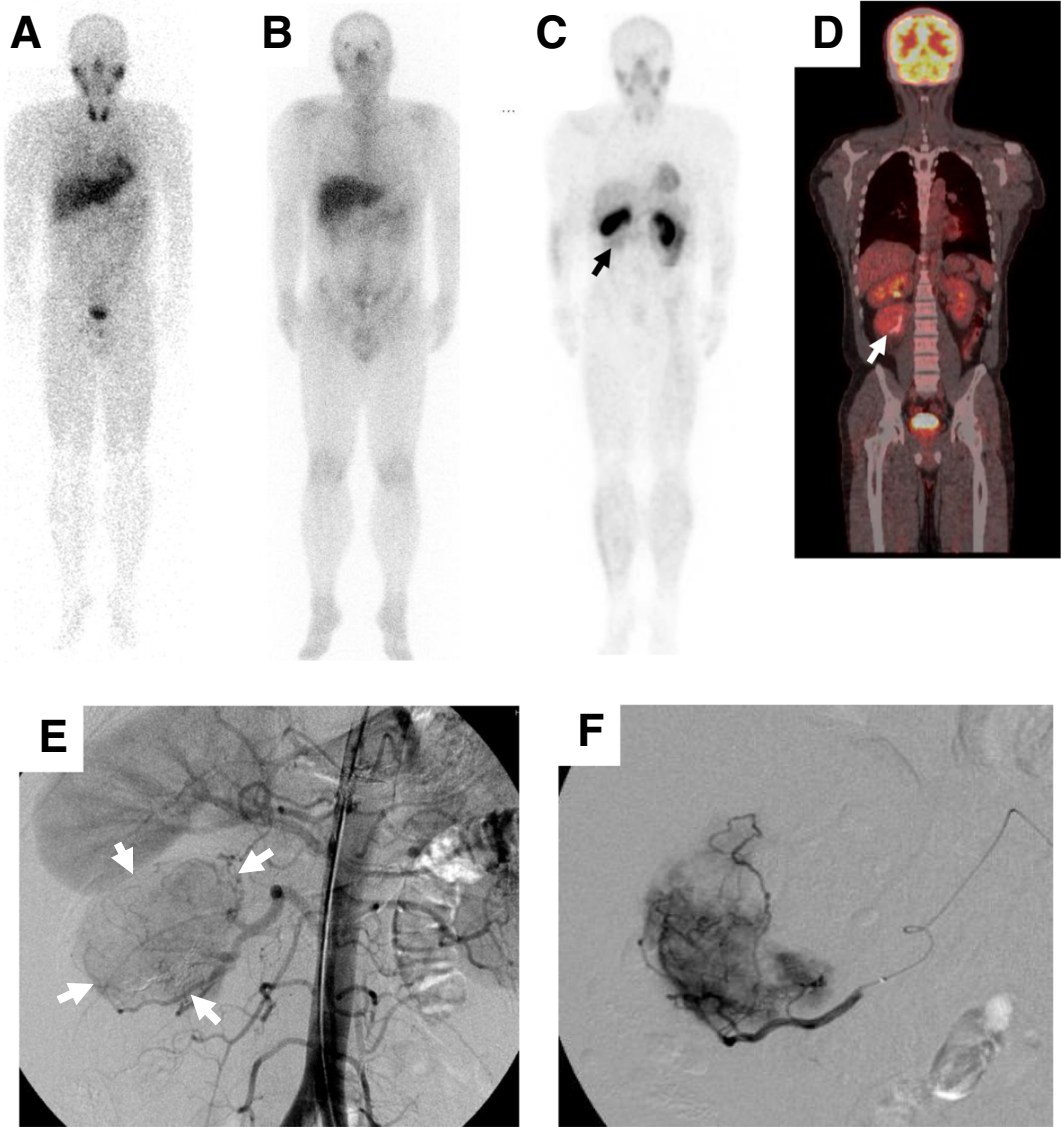
**Radionuclide imaging and angiography.** Both iodine-123 metaiodobenzylguanidine (**A**) and gallium-67 (**B**) scintigraphies were negative in the mass, whereas thallium-201 mildly accumulated in the tumor below the right kidney (**C**, arrow). Positron emission tomography was positive in the tumor at a standard uptake value of 4.7 without any other abnormal uptake in other parts of the body (**D**). Digital subtraction angiography revealed abundant vascularity of the tumor (**E**, arrow). Feeding vessels from the right lumbar arteries were embolized with porous gelatin particles (**F**).

A 33-year-old man with epigastric discomfort underwent abdominal ultrasonic examination, revealing a solid mass next to the right kidney. Abdominal radiography revealed striated calcification on the right side of the first and second lumbar vertebrates (Figure 1A,B). Computed tomography (CT) demonstrated a well-circumscribed, homogenous mass with central calcification in the right psoas muscle (Figure 1C). Characteristically, the mass presented in a dumbbell-like shape, extending to the intervertebral foramen on contrast-enhanced CT (Figure 1D). On the basis of the characteristic shape, a neurogenic tumor was suspected. Magnetic resonance imaging (MRI) revealed an isointense mass on the T1-weighted image (T1WI) and a hyperintense mass on the T2-weighted image (T2WI) with a central radial hypointense area (Figure 2A,B). On the caudal and anterior side of the tumor, a lipomatous component was observed (Figure 2B,C). Contrast-enhanced axial T1WI showed marked enhancement of the tumor (Figure 2D). Both iodine-123 metaiodobenzylguanidine and gallium-67 scintigraphies were negative in the mass, suggesting pheochromocytoma and lymphoma as less susceptive diagnoses (Figure 3A,B). Thallium-201 mildly accumulated in the tumor, suggesting blood flow to the tumor (Figure 3C). Positron emission tomography (PET) revealed accumulation of fluorine-18-2-fluoro-2-deoxy-d-glucose in the tumor at a standard uptake value (SUV) of 4.7, whereas no other abnormal uptake suggestive of a metastatic lesion was demonstrated (Figure 3D). On the basis of all imaging findings, preoperative differential diagnoses included schwannoma, inflammatory myofibroblastic tumor, and liposarcoma. Because significant blood flow to the tumor was suspected, angiography was performed on the day prior to surgery revealing abundant vascularity (Figure 3E). Feeding vessels from the right lumbar arteries were embolized with porous gelatin particles (Gelpart, Nihon-Kayaku, Tokyo, Japan) in order to reduce intraoperative bleeding (Figure 3F).

**Figure 4:**
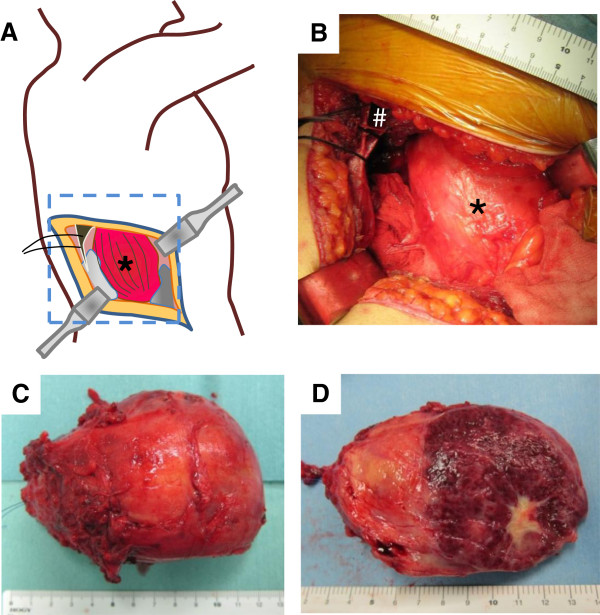
**Intraoperative gross findings.** Tumor resection was performed with the retroperitoneal approach from the right side by resecting the 11^th^ rib (**A**,**B**). The tumor existed in the swollen psoas muscle (*), adjacent to the posterior-distal edge of the pleural cavity (**B**, **#**). The tumor was an encapsulated, tan, elastic soft oval mass sized 11 × 8 × 8 cm (**C**). The cut surface was dark red with a central white zone of fibrosis and calcification (**D**).

Surgical resection was performed with the retroperitoneal approach from the right side by resecting the 11^th^ rib (Figure 4A). The tumor existed in the swollen psoas muscle, accompanying multiple feeding vessels around the tumor (Figure 4B). Before excision, a small piece of tumor tissue was submitted for intraoperative pathological examination by frozen section. The pathological examination was suggestive of inflammatory myofibroblastic tumor, and no evidence of malignancy was noted. We decided to perform marginal excision of the tumor. After ligation and transection of several feeding vessels, we carefully dissected the tumor that adhered tightly to surrounding muscle tissues. The total blood loss was 940 g and the patient recovered without any postsurgical complication. The tumor was an encapsulated, tan, elastic soft oval mass sized 11 × 8 × 8 cm (Figure 4C). The cut surface was dark red with a central white zone of fibrosis and calcification (Figure 4D).

**Figure 5:**
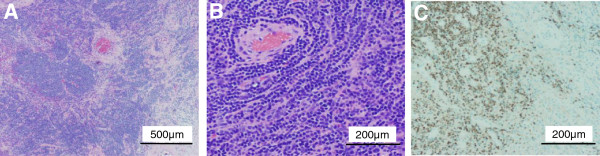
**Pathological findings.** Lymphocytes formed lymphoid follicles and capillary proliferation was observed (**A**). Capillaries were surrounded by hyaline sheaths (**B**). Immunostaining revealed that the proliferated lymphocytes were positive for the B-cell marker CD79a (**C**). The diagnosis of CD of hyaline-vascular type was established.

Pathological examination demonstrated infiltration of lymphocytes forming lymphoid follicles accompanied with capillary proliferation (Figure 5A). Capillaries were surrounded by hyaline sheaths (Figure 5B). Immunostaining revealed that the proliferated lymphocytes were positive for the B-cell marker CD79a and negative for the T-cell marker CD3 (Figure 5C). In general, abundant IgG4-positive plasma cells are observed in plasma cell type CD, whereas hyaline-vascular type CD is accompanied only by a few IgG4-positive plasma cells [8]. IgG4-positive cells were very sparsely observed in the present case, ruling out plasma cell type CD. The diagnosis of hyaline-vascular type UCD (HV-UCD) was established.There was no sign of recurrence at 1 year after surgery.

## Discussion

A recent meta-analysis of retroperitoneal tumors showed that sarcoma comprise a third of retroperitoneal tumors [9]. A variety of neoplasms could occur in the retroperitoneal space, including liposarcoma, lymphoma, epithelial tumors, or metastatic disease from known or unknown primary sites. Benign retroperitoneal tumors include benign neurogenic tumors, paragangliomas, fibromatosis, renal angiomyolipomas, and benign retroperitoneal lipomas. Because of the late presentation of symptoms, tumors have often reached a significant size. Therefore, surgical resections are often incomplete, resulting in local recurrence associated with a less favorable outcome. A preoperative diagnosis is important in order to plan the surgical procedure and adjuvant treatment. However, because of the rarity of the disease, a preoperative diagnosis of UCD was not made in previous case reports of UCD [10,11], resulting in the surgical exploration of suspicious malignant tumors, including liposarcomas [12], malignant fibrous histiocytomas [7], vascular sarcomas [13], and adrenal neoplasms [14]. Retroperitoneal schwannoma is also rare, but anatomical proximity to adjacent neural structures in imaging studies should raise suspicion [15]. In the present case, the tumor was located in the psoas muscle, and the medial edge of the tumor extended towards the lumbar intervertebral foramen as demonstrated by CT and MRI (Figures 1 and 2) suggesting schwannoma as primary suspicious diagnosis. A characteristic imaging finding of schwannoma is the target sign, which histologically corresponds to peripheral myxomatous tissue and central collagenous tissue [16]. The present case also showed the target-like pattern in T2WI of MRI, surrounded by fat tissue (Figure 2B). The central area of low intensity histologically corresponded to fibrous tissue (Figure 5), which could be found in abdominal CD. However, Zhou et al. reported that a central area of low-density on CT and low-intensity on MRI showed a fissured and radial pattern [17], which was found in our case. Therefore, the presence of the target sign on MRI as well as the shape of the central low-intensity area may help to distinguish CD from schwannoma.

Because the findings by CT and MRI did not lead to a definite preoperative diagnosis, radionuclide studies, including PET, were performed. Negativity of gallium scintigraphy suggested malignant lymphoma as a less suspected diagnosis. It is reasonable that CD could be positive for gallium scintigraphy because of its nature of lymphatic proliferative disease [18]. However, its positivity would depend on the inflammatory activity of an individual case, which explains why the present case was negative for gallium-67. PET recently became a common modality for the diagnosis and evaluation of therapeutic effects on malignant tumors. A recent study revealed that the median SUV of seven patients with active MCD was 4.8 (range, 2.6-9.3) [19], which was comparative to that of the present case (SUV, 4.7). High cellularity and inflammatory nature of CD may lead to high SUV, making it difficult to exclude malignant tumor before biopsy. Oida et al. reported a case of UCD that showed an SUV of 4.5 in the PET study, resulting in its incorrect preoperative diagnosis as lymphoma [20]. Therefore, even with the most advanced imaging techniques, including PET, the preoperative diagnosis of retroperitoneal tumors, including CD, still seems difficult [2].

HV-UCD is the most common variant of CD and accounts for 72% of all CD variants [3]. Plasma cell type CD (PC-UCD) displays of mature plasma cell proliferation in the areas surrounding the germinal centers, without accompanyinghyalinized vessel formation [3,8]. PC-UCD and PC-MCD account for 18% and 10% of all PC variants, respectively. The treatment differs by the type of CD, reflecting the clinical behavior of each variant. Both HV-UCD and PC-UCD can be cured by complete resection of the affected lymph node; recurrence, metastasis, or mortality have not been reported [2,3,21]. Rare cases of PC-UCD show remnant systemic symptoms after resection; however, additional therapy for those PC-UCD cases is not well established [3]. Radiation therapy may be a treatment option for cases of surgically unresectable UCD [2,21]. Cure can be achieved by radiotherapy in selected patients with UCD, although its role in UCD treatment remains unclear [21]. Regarding the follow-up for UCD, patients without systemic involvement should have an additional radiological assessment 6–12 months postoperatively to verify the cure; additional testing or therapy should be considered at the onset of new symptoms [10].

Surgeons should be prepared for massive bleeding during the resection of CD, especially in the deep portion of the body close to major vessels. Preoperative angiography clearly visualized the feeding vessels that aided intraoperative detection and ligation of the feeding lumbar artery. If such an imaging study is not performed and the preoperative diagnosis does not include such hemorrhagic tumors, massive bleeding may obstruct surgical procedures and patients may be at risk of fatal blood loss. Although we chose the retroperitoneal approach in the decubitus position in the present case, the transperitoneal approach may have been an alternative option for tumor resection. One of the reasons for us choosing this is approach is familiarity with the retroperitoneal approach for spinal surgery. Another reason is that exploration of the intervertebral foramen may have been required because schwannoma was the most suspected preoperative diagnosis. If UCD is preoperatively suspected and the surgeons are familiar with the transperitoneal approach, the wide operative field obtained with that approach may be helpful to reduce intraoperative bleeding.

## Conclusions

The paraspinal mass that resembled a schwannoma proved to be a rare case of UCD. Careful preoperative assessment of the imaging study enables proper planning of the surgery. More importantly, central low areas in fissured and radial pattern on CT or MRI would be helpful for making the correct diagnosis of CD.

## Consent

Written informed consent was obtained from the patient for publication of this Case report and any accompanying images. A copy of the written consent is available for review by the Series Editor of this journal.

## Abbreviations

CD: Castleman’s disease; UCD: Unicentric CD; MCD: Multicentric CD; HV-UCD: Hyaline-vascular type UCD; PC-UCD: Plasma cell type CD; SUV: Standard uptake value; T1W1: T1-weighted image; T2W1: T2-weighted image

## Competing interests

The authors declare that they have no competing interests.

## Authors’ contributions

SN, MY, and TY participated in the surgical treatment and follow-up of the patient. HK and SK helped draft and finalize the manuscript. TH, YT, and SY performed the pathological examination. All authors read and approved the manuscript.
